# Evidence for proposed ICD-11 PTSD and complex PTSD: a latent profile analysis

**DOI:** 10.3402/ejpt.v4i0.20706

**Published:** 2013-05-15

**Authors:** Marylène Cloitre, Donn W. Garvert, Chris R. Brewin, Richard A. Bryant, Andreas Maercker

**Affiliations:** 1 National Center for PTSD, Veterans Affairs Palo Alto Health Care System, Palo Alto, CA, USA; 2 Department of Psychiatry and Child and Adolescent Psychiatry, New York University, Langone Medical Center, New York, NY, USA; 3 University College London, UK; 4 School of Psychology, University of New South Wales, Sydney, New South Wales, Australia; 5 Department of Psychopathology and Clinical Intervention, University of Zurich, Switzerland

**Keywords:** Complex PTSD, posttraumatic stress disorder, WHO, ICD-11

## Abstract

**Background:**

The WHO International Classification of Diseases, 11th version (ICD-11), has proposed two related diagnoses, posttraumatic stress disorder (PTSD) and complex PTSD within the spectrum of trauma and stress-related disorders.

**Objective:**

To use latent profile analysis (LPA) to determine whether there are classes of individuals that are distinguishable according to the PTSD and complex PTSD symptom profiles and to identify potential differences in the type of stressor and severity of impairment associated with each profile.

**Method:**

An LPA and related analyses were conducted on 302 individuals who had sought treatment for interpersonal traumas ranging from chronic trauma (e.g., childhood abuse) to single-incident events (e.g., exposure to 9/11 attacks).

**Results:**

The LPA revealed three classes of individuals: (1) a complex PTSD class defined by elevated PTSD symptoms as well as disturbances in three domains of self-organization: affective dysregulation, negative self-concept, and interpersonal problems; (2) a PTSD class defined by elevated PTSD symptoms but low scores on the three self-organization symptom domains; and (3) a low symptom class defined by low scores on all symptoms and problems. Chronic trauma was more strongly predictive of complex PTSD than PTSD and, conversely, single-event trauma was more strongly predictive of PTSD. In addition, complex PTSD was associated with greater impairment than PTSD. The LPA analysis was completed both with and without individuals with borderline personality disorder (BPD) yielding identical results, suggesting the stability of these classes regardless of BPD comorbidity.

**Conclusion:**

Preliminary data support the proposed ICD-11 distinction between PTSD and complex PTSD and support the value of testing the clinical utility of this distinction in field trials. Replication of results is necessary.

The World Health Organization (WHO) is responsible for developing the International Classification of Diseases, 11th version (ICD-11), which is expected to be completed in 2015. Within the spectrum of stress and trauma disorders, the WHO ICD-11 has proposed two related diagnoses, posttraumatic stress disorder (PTSD) and complex PTSD (Maercker et al., 2013). WHO has emphasized clinical utility as the organizing principle in classification development. This means that diagnoses should be consistent with clinicians’ mental health taxonomies, limited in number of symptoms, and based on distinctions important for management and treatment (Reed, 2010). These recommendations guided the organization of the PTSD and complex PTSD diagnoses as well as their relationship to each other. This study provides the first empirical support for a separation of these two conditions.

The proposed distinction conforms to the ICD-11 goal of clinical utility by virtue of its relative simplicity in the classification structure, clear differences in conceptual organization and limited set of symptom features. In the proposed ICD-11 hierarchical classification structure, PTSD and complex PTSD are “sibling” disorders, meaning that the diagnoses follow from the parent category of traumatic stress disorders. The stressor acts as the “gate” which allows consideration of a diagnosis of either PTSD or complex PTSD (see Table 1). Regardless of the nature of the stressor, the diagnosis of PTSD or complex PTSD is determined by the symptom profile. This simplifies the task of diagnosis for the clinician by focusing on the target of treatment, namely symptoms and problems, rather than on trauma history.

**Table 1:** PTSD and complex PTSD in classification hierarchy

Traumatic stress disorders	
“Gate” criterion: traumatic stressor	
Select either PTSD or complex PTSD	
	
PTSD	Complex PTSD
Re-experiencing	Re-experiencing
Avoidance	Avoidance
Sense of threat	Sense of threat
	Affect dysregulation
	Negative self-concept
	Interpersonal disturbances

The two disorders have distinct but related conceptual frames that organize the symptom picture. The PTSD diagnosis is proposed to consist of a reduced set of six symptoms making up three core elements, each of which is required for the diagnosis: re-experiencing of the traumatic event(s) in the present accompanied by emotions of fear or horror; avoidance of traumatic reminders; and a sense of current threat that is manifested by excessive hypervigilance or an enhanced startle reaction. The syndrome has fear or horror at its heart with a focus on the re-experiencing of the trauma memory and consequent avoidance and hypervigilance. This formulation conceptualizes PTSD essentially as a fear condition and emphasizes symptoms that distinguish it from other psychiatric disorders, in line with recommendations by Brewin, Lanius, Novac, Schnyder, and Galea (2009) and Spitzer, First, and Wakefield (2007).

Proposed ICD-11 complex PTSD is a disorder that requires PTSD symptoms as defined above but also includes three additional features that reflect the impact that trauma can have on systems of self-organization, specifically problems in affective, self-concept, and relational domains. Unlike the PTSD symptoms in which reactions of fear or horror are tied to trauma-related stimuli, these three latter types of disturbances are pervasive and occur across various contexts and relationships regardless of proximity to traumatic reminders. The proposal for a second trauma-related disorder was first articulated by Herman (1992) who described the potential impact of prolonged traumatic stressors (e.g., torture, domestic violence, childhood abuse) on self-organization, independent of PTSD symptoms. This conceptualization of complex PTSD was operationalized under the name Disorders of Extreme Stress Not Otherwise Specified (DESNOS) for the DSM-IV field trials (see Roth, Newman, Pelcovitz, Van der Kolk, & Mandel, 1997). Findings from the DSM-IV field trial revealed high rates of endorsement of symptoms representative of disturbances in affective, self, and relational domains among those with chronic trauma compared to participants with other types of trauma histories. The selection of specific symptoms representative of the above domains for the ICD-11 proposal was guided by the symptoms most frequently reported by participants in the DSM-IV field trials (see Van der Kolk, Roth, Pelcovitz, Sunday & Spinazzola, 2005) as well those identified as most frequent and most impairing by expert clinicians in a recent consensus survey on complex PTSD (Cloitre et al., 2011). Notably, the data from the DSM-IV field trials showed that nearly all of those who meet criteria for DESNOS also meet criteria for PTSD (Roth et al., 1997), supporting the proposed ICD-11 formulation of complex PTSD, which incorporates PTSD symptoms as a core component.

In the proposed ICD-11, diagnosis of complex PTSD requires the presence of PTSD as well as the presence of at least one symptom in each of three self-organization features (affect, negative self-concept and relational disturbance). The affective domain problems are characterized by emotion dysregulation as evidenced by heightened emotional reactivity, violent outbursts, reckless or self-destructive behavior, or a tendency towards experiencing prolonged dissociative states when under stress. In addition, there may be emotional numbing and a lack of ability to experience pleasure or positive emotions. Self-disturbances are characterized by negative self-concept marked by persistent beliefs about oneself as diminished, defeated or worthless. These can be accompanied by deep and pervasive feelings of shame or guilt related to, for example, not having overcome adverse circumstances, or not having been able to prevent the suffering of others. Interpersonal disturbances are defined by persistent difficulties in sustaining relationships. These difficulties may present in a variety of ways but are exemplified by difficulties in feeling close to others. Individuals may consistently avoid, deride or have little interest in relationships and social engagement more generally. The person may occasionally experience close or intense relationships but will have difficulty maintaining emotional engagement.

The disturbances in self-organization are proposed to be associated with, although not a necessary consequence of, sustained exposure to repeat or multiple types of traumatic stressors (e.g., childhood abuse, domestic violence, genocide campaigns, torture). Indeed, the association between exposure to sustained traumatic stressors and disturbances in affect, self-concept, and relational difficulties has been supported in the literature (Briere & Rickards, 2007). However, ICD-11 proposes the association with multiple stressors as a risk factor for, rather than requirement of, the disorder. This guideline recognizes the role of genetic and environmental factors that may influence the relationship between events and psychological consequences. It allows the designation of complex PTSD related to a single-incident stressor in a vulnerable person and, conversely, the designation of PTSD or no disorder in a resilient person who has experienced prolonged and repeated exposure to traumatic stressors.

Investigation of the actual clinical utility of this proposal via field trials evaluating the clarity and ease of differential diagnosis between the two disorders among community clinicians is ongoing. However, empirical support for the distinction between PTSD and complex PTSD based on the symptoms of trauma-exposed samples is needed. This article reports on the results of an initial investigation of the validity of the two constructs by using latent profile analysis (LPA). LPA is a form of multivariate analysis that can identify subgroups of individuals who are empirically distinguishable based on different patterns of symptom endorsements (Lazarsfeld & Henry, 1968). Accordingly, we hypothesized that the analyses should identify at least two classes of individuals: one characterized by elevations on the PTSD symptoms but not on the affect, negative self-concept, or interpersonal symptoms (PTSD) and the second characterized by elevations on the PTSD symptoms, as well as on affect, self-concept, and interpersonal symptoms (complex PTSD).

We also conducted two additional tests of differences between the proposed disorders. Consistent with the literature cited above, we hypothesized that sustained exposure to repeat or multiple types of traumatic stressors would be a greater risk factor for complex PTSD than PTSD; conversely, single-event traumatic stressors would be a greater risk factor for PTSD than complex PTSD. We also hypothesized that complex PTSD would be associated with more severe functional impairment than PTSD and that the symptom domains of affective, self-concept, and interpersonal disturbance would contribute significantly to the overall impairment above and beyond those contributed by the PTSD symptoms.

Finally, a recent review (Resick et al., 2012) has identified a potential symptom overlap such that the complex PTSD symptom set may not represent a distinct diagnosis but rather the presence of PTSD comorbid with borderline personality disorder (BPD). Because our goals were to evaluate whether, in fact, complex PTSD stands on its own as a coherent and distinct construct and to identify sociodemographic, trauma history and clinical characteristics associated with this disorder and not BPD, we removed all participants with BPD from our primary data analyses. Under this condition, a confirmatory factor analysis would provide a test of whether the data factored as predicted by the proposed structure of the complex PTSD diagnosis, independent of the BPD construct. Similarly, the LPA would test for the presence of a distinct subgroup of the individuals that endorsed both the PTSD and self-organizational difficulties but did not have BPD. Finally, in order to assess the impact of BPD on our primary hypothesis, namely, the presence of PTSD and complex PTSD as representative of distinct classes of individuals, we conducted a second LPA that included individuals with BPD, to identify potential changes in the organization of the classes and in the pattern and severity of symptoms.

## Methods

### Participants and procedures

The data for these analyses were obtained from an archival data set of measures completed as part of the routine assessment of all individuals seeking treatment at a New York City trauma clinic during the years 2002–2007 (*n*=388). For problems related to interpersonal violence. Traumas for which individuals sought treatment included childhood sexual abuse, childhood physical abuse, adulthood sexual assault, and adulthood physical assault, abuse, and mass violence. Due to the attack on the World Trade Center on September 11, 2001, there was a substantial flow of individuals seeking treatment for 9/11-related problems. Trauma survivors were self-referred by means of advertisements in the community or word-of-mouth or clinicians for various clinical trials and research treatment studies. A total of 86 (22.2%) participants were identified as having BPD.

After removing participants with BPD, a total of *n=*302 remained. Participants had a mean age of 39.57 (*SD*=11.53) years. The majority of the sample was female (89.1%, *n*=269). The majority of the sample identified as Caucasian (53.3%, *n*=161), followed by African–American (18.2%, *n*=55), Hispanic (16.2%, *n*=50), others (8.3%, *n*=25), Asian (2.6%, *n*=8), and unknown (1.0%, *n*=3). Marital status was as follows: 48.3% (*n*=146) reported being single, married (18.2%, *n*=55), divorced or separated (16.2%, *n*=49), living with a significant other (14.9%, *n*=45), widowed (1.7%, *n*=5), and unknown (0.7%, *n*=2). More than half of the sample endorsed completing some or having graduated from college (58.6%, *n*=177) or attaining education post-college (30.8%, *n*=93). The majority of participants reported some employment with 46.4%, (*n*=140) being employed full-time (35 h and above per week) and 16.6% (*n*=50) indicating they were employed at least part-time (<35 h per week).

Frequency of interpersonal violence traumas were as follows: childhood sexual abuse (53.0%), childhood physical abuse (58.6%), childhood sexual assault (a single incident by stranger or non-caretaker) (13.2%), adulthood sexual assault (37.4%), adulthood physical assault (32.1%), and 9/11 (28%). Additional frequently reported traumas included: sudden traumatic death of someone close due to murder, suicide, accident, or medical illness (63.6%), being in a serious accident (38.7%), and being in a disaster (34.8%). Among these, the most frequently identified as the “worst trauma” were childhood sexual or physical abuse (30.1%), 9/11 (19.9%) and sudden traumatic death of someone close (10.3%).

### Measures

Trauma history was determined using the Life Events Checklist, a 23-item questionnaire adapted from the Life Stressor Checklist-Revised (Wolfe & Kimerling, 1997) to include questions concerning childhood abuse, sexual assault and mass violence (including 9/11). It is clinician administered and includes queries of age at the time of event and identification of “worst trauma.” Identification of BPD was made using the DSM-IV SCID II (First, Gibbon, Spitzer, Williams, & Benjamin, 1997), where positive BPD status was defined as endorsement of five or more symptoms. Two measures were used for the PTSD and complex PTSD symptoms sets: the Modified PTSD Symptom Scale—Self-Report Severity (MPSS-SR; Falsetti, Resnick, Resick, & Kilpatrick, 1993) and the Brief Symptom Inventory (BSI; Derogatis & Melisaratos, 1983). Functional impairment was assessed with the Social Adjustment Scale—Self-Report (SAS-SR; Weissman & Bothwell, 1976). The MPSS-SR provided the reduced PTSD symptom set as they are specified for ICD-11 (see Van Emmerik & Kamphuis, 2011). The complex PTSD construct items were selected from a combination of the MPSS-SR and the BSI, which measures a wide range of psychiatric symptoms. Items used to evaluate the complex PTSD construct were selected based on face validity (directly or closely representative of the symptoms). The items used to represent the symptoms of PTSD and complex PTSD are shown in Table 2.

**Table 2:** Items representing PTSD and complex PTSD

Factor	Cluster	Test Items
PTSD	Re-experiencing	MPSS-SR 2. Having bad dreams or nightmares about the trauma
		MPSS-SR 3. Reliving the trauma, acting or felling as if it were happening again
	Avoidance	MPSS-SR 5. Trying not to think about, talk about or have feelings about the trauma
		MPSS-SR 6. Trying to avoid activities, people or places that remind you of the trauma
	Sense of threat	MPSS-SR15. Being over alert (for example, checking to see who is around you, being uncomfortable with your back to the door)
		MPSS-SR16. Being jumpy or easily startle (for example, when someone walks up behind you)
Affect dysregulation		BSI 13. Temper outbursts that you could not control
		BSI 20. Your feelings easily hurt
Negative self-concept		BSI 50. Feelings of worthlessness
		BSI 52. Feelings of guilt
Interpersonal problems		BSI 44. Never feeling close to another person
		MPSS-SR 9. Feeling distant or cut off from other people

#### MPSS-SR

The MPSS-SR is a brief self-report instrument which assesses the severity of each of the 17 PTSD symptoms outlined in the DSM-IV on a 5-point Likert scale ranging from 0=*not at all* to 4=*extremely*. The MPSS-SR has demonstrated excellent psychometric properties (Falsetti et al., 1993). In our sample, the MPSS-SR demonstrated good internal consistency (*α*=0.89).

#### BSI

The BSI is a 53-item self-report psychological symptom inventory with nine primary symptom dimensions. The measure assesses how much a problem bothered or distressed a person using a 5-point Likert scale ranging from 0=“not at all” to 4=“extremely”. The BSI has shown high convergent and construct validity (Derogatis & Melisaratos, 1983). In our sample, the BSI demonstrated excellent internal consistency (*α*=0.96).

#### SAS-SR

The Social Adjustment Scale-Self Report (SAS-SR; Weissman & Bothwell, 1976) was used to measure functional impairment. The SAS-SR consists of 42 Likert-type items, which assess the level of functioning over the past two weeks for six domains: work, social and leisure activities, relationships with extended family, role as a marital partner, parental role, and role within the family unit. A mean score can be calculated for each of the six domains, as well as one overall mean score, based on the total number of items relevant to the responder. Higher scores indicate greater impairment. The SAS-SR has demonstrated strong psychometric properties among community and clinical samples (e.g., Weissman & Bothell, 1976).

### Statistical analyses

#### Confirmatory factor analysis

The model analyzed for this study was a four-factor model, which was composed of the following factors: PTSD, affect dysregulation, negative self-concept, and interpersonal problems. All items used in the confirmatory factor analysis (CFA) were standardized prior to performing the CFA. The PTSD factor included the three core elements (re-experiencing, avoidance, and sense of threat), and each element was composed of two symptoms as specified in the ICD-11 proposal. The model allowed the error terms for the respective pairs of items for each symptom to correlate. The three self-regulation factors (affect dysregulation, negative self-concept, and interpersonal problems) were operationalized using only two items, therefore a constraint was used in these factors, where the loadings of both items were set equal to 1.00 in order to make the model just identified. The fit of the model was assessed using the following fit indices: comparative fit index (CFI), Tucker–Lewis index (TLI), and the root mean-square error of approximation (RMSEA). Recent literature has suggested that CFI ≥0.95, TLI ≥0.95, and RMSEA ≤0.06 are indicative of a strong model fit (Kline, 2004).

#### Latent profile analyses

The optimal number of classes was evaluated using the Lo–Mendell–Rubin-adjusted likelihood ratio test (LMR-A), as well as the bootstrap likelihood ratio test (BLRT) and the Bayesian Information Criterion (BIC), both of which have been shown to be very consistent indicators of classes (Nylund, Asparouhov, & Muthen, 2007). The general practice of LPA is to test the fit of a two-class model and systematically increase the number of classes until adding more classes is no longer warranted. The LMR-A compares the fit of the specified class solution to models with one less class. A *p*-value<0.05 suggests that the specified model provides a better fit to the data relative to the model with one less class. Similarly, a statistically significant BLRT suggests that the current model is preferred over a model with one less class. The BIC provides information about model fit with lower relative values indicating improved model fit. The 12 standardized items that were used in the CFA were also used in the LPA.

#### Descriptive and regression analyses

ANOVAs were performed to assess differences in sociodemographic characteristics, trauma history, and symptom severity across the classes identified in the LPA. Logistic regression analyses were performed to determine the predictive value that different kinds of trauma, particularly chronic repeated trauma, such as childhood abuse, and single adult trauma, such as 9/11, had in predicting membership in the different classes. A hierarchical linear regression analysis was performed to evaluate the relative contributions of the four core elements of complex PTSD (PTSD, affect dysregulation, negative self-concept, and interpersonal problems) to functional impairment.

## Results

### Complex PTSD confirmatory factory analysis

**Fig. 1:**
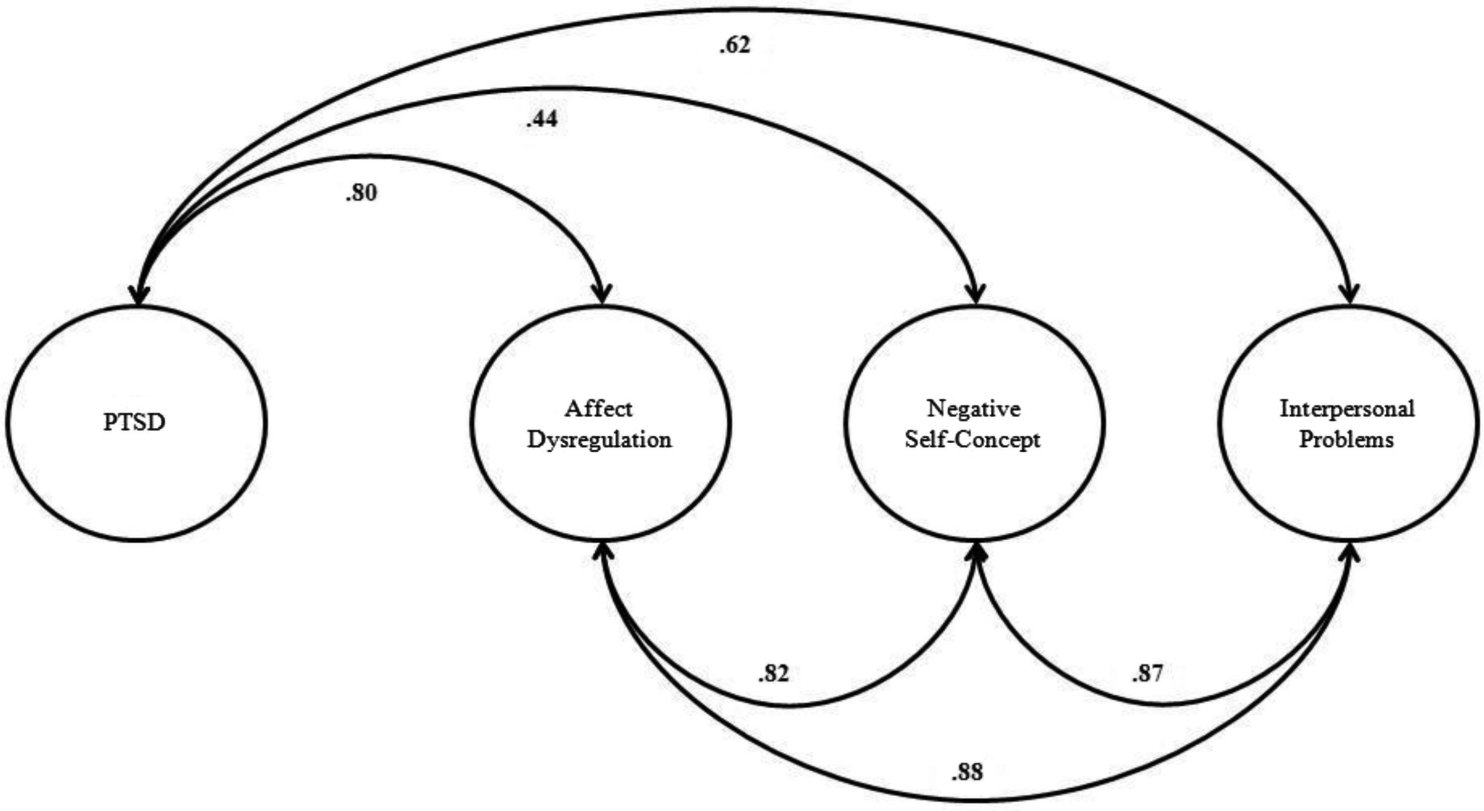
Factor correlations of the four-factor model of complex PTSD.

The fit of the four-factor model of complex PTSD was strong, as it yielded a CFI=0.97, TLI=0.96, and RMSEA=0.05 (90% CI: 0.03, 0.07). The correlations between the four-factors of complex PTSD are shown in Fig. 1. The factors unique to complex PTSD were more highly correlated with each other (*r*=0.82–0.88) than to the PTSD factor; however, the relationship of each to the PTSD factor was moderate to strong (*r*=0.44–0.80).

### Latent profile analysis

The two- and three-class models both yielded a significant LMR-A and BLRT result at *p*<0.05. Since the four-, five-, and six-class models did not have a significant LMR-A, they were not considered for the final model. The three-class model was selected over the two-class model as the BIC value was lower in the three-class model. The fit indices of the different class models are shown in Table 3.

**Table 3:** Latent profile models and fit indices

Model	Log-likelihood	BIC	Entropy	LMR-A *p*-value	BLRT *p*-value
2 classes	−4777.98	9767.24	0.87	<0.001	<0.001
3 classes	−4673.08	9631.68	0.85	0.004	<0.001
4 classes	−4592.53	9544.81	0.86	0.267	<0.001
5 classes	−4551.79	9537.57	0.88	0.158	<0.001
6 classes	−4515.36	9538.95	0.87	0.728	<0.001^a^

*Note:* BIC, Bayesian information criterion; LMRA-A, Lo-Mendell-Rubin adjusted likelihood ratio test; BLRT, bootstrap likelihood ratio test.

a The best log-likelihood value was not replicated in 31 out of 50 bootstrap draws. The *p*-value may not be trustworthy due to local maxima.

**Fig. 2:**
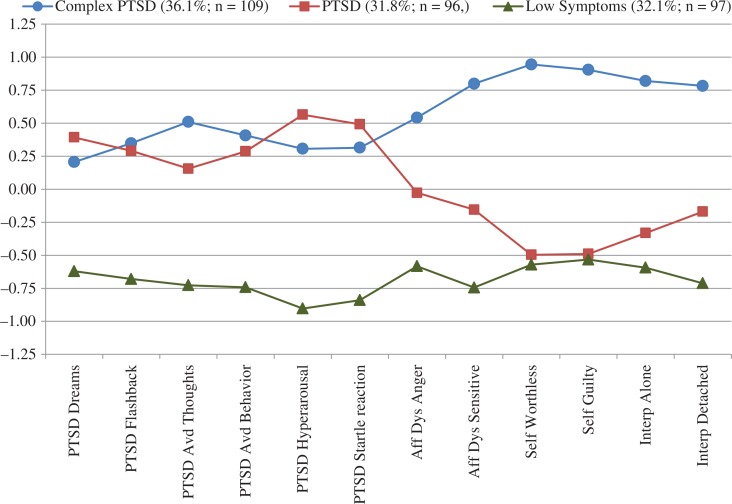
Mean standardized values of complex PTSD items.

The three classes were compared on the 12 standardized items that were used to determine class membership in order to provide descriptive labels of the different classes. Class 1 was labeled as “complex PTSD” as this class had high levels of symptoms in PTSD, affect dysregulation, negative self-concept, and interpersonal problems items. Class 2 was labeled as “PTSD” as this class had high levels of PTSD symptoms but relatively low levels of symptoms in the three self-organization domains. Class 3 was labeled as “low symptom” as this class had relatively low levels of all symptoms. The mean standardized values of the items by class are shown in Fig. 2.

The mean probability of class membership in the three-class model was acceptable: 0.96 for the complex PTSD class, 0.89 for the PTSD class, and 0.95 for the low symptom class, which implies acceptable discrimination among the classes. An acceptable entropy value probability of 0.85 lends support to this result by suggesting adequate latent class separation. Overall, 36.1% (*n*=109) of participants were classified into complex PTSD class, 31.8% (*n*=96) into the PTSD class, and 32.1% (*n*=97) into the low symptom class.

### Sociodemographic, trauma history, and symptom characteristics

The three classes did not differ by age, gender, ethnicity or employment status. Differences in type of trauma (see Table 4) emerged such that the PTSD class more frequently endorsed 9/11 as their worst trauma as compared to the other two groups, while the complex PTSD group tended to more frequently endorse childhood (sexual and/or physical) abuse as the worst trauma compared to the PTSD group (*p*<0.07). The cumulative number of different types of childhood interpersonal violence traumas (sexual abuse, physical abuse, and childhood sexual assault) was higher in the complex PTSD class than the other two classes. The number of adulthood interpersonal violence traumas was higher in the two diagnostic classes as compared to the “low symptom” class. The three classes did not differ in the total number of different types of trauma. As indicated in Table 5, the complex PTSD class did not differ from the PTSD class on severity of PTSD symptoms but was higher than the other two classes in affect dysregulation, negative self-concept, and interpersonal problems as well as in functional impairment.

**Table 4:** Demographic and trauma characteristics of the three classes

Characteristics	Class 1 Complex PTSD *n*=109	Class 2 PTSD *n*=96	Class 3 Low symptom *n*=97	Significance test
Age (M (SD))	39.47 (11.24)	39.18 (11.44)	40.07 (12.03)	NS
Female	91.7%	89.6%	85.6%	NS
Ethnicity (% white)	49.5%	52.7%	59.8%	NS
Employed (full or part-time)	63.0%	61.7%	66.7%	NS
9/11 exposure	27%	44%	40%	p =0.03 2, 3>1
9/11 was worst trauma	12.4%	36.4%	17.6%	*p*<0.001 2>1, 3
Childhood abuse^a^	87.6%	74.2%	73.9%	*p*=0.024 1>3
Childhood abuse^a^ was worst trauma	41.2%	25.0%	34.1%	*p*=0.070 1>2
Any childhood interpersonal violence^b^	89.6%	77.8%	76.1%	*p*=0.026 1>3
Any adulthood interpersonal violence^c^	65.4%	64.9%	46.3%	*p*=0.008 1, 2>3
Childhood abuse total^a^	1.36 (0.70)	1.07 (0.77)	1.09 (0.79)	*p*=0.012 1>2, 3
Childhood interpersonal violence total^b^	1.52 (0.80)	1.21 (0.87)	1.18 (0.85)	*p*=0.008 1>2, 3
Adulthood interpersonal violence total^c^	0.83 (0.71)	0.77 (0.66)	0.51 (0.58)	*p*=0.001 1, 2>3
All events total^d^	3.66 (1.67)	3.38 (1.59)	3.16 (1.57)	NS

a Childhood abuse=sexual and/or physical abuse

b childhood interpersonal violence=sexual abuse, physical abuse, childhood sexual assault

c adult interpersonal violence=sexual assault or physical assault

d all events total score is based on 8 possible events=childhood sexual abuse, childhood physical abuse, childhood sexual assault, adult sexual assault, adult physical assault, sudden death of someone close, being in an accident, and being in a disaster.

**Table 5:** Symptom characteristics of the three classes

Characteristics	Class 1 Complex PTSD *n*=109	Class 2 PTSD *n*=96	Class 3 Low symptom *n*=97	Significance test
PTSD	14.60 (4.94)	14.71 (3.47)	5.34 (3.09)	*p*<0.001 1, 2>3
Re-experiencing	3.50 (2.47)	3.68 (2.10)	0.99 (1.29)	*p*<0.001 1, 2>3
Avoidance	5.93 (2.09)	5.25 (2.08)	2.48 (2.22)	*p*<0.001 1, 2>3
Sense of threat	5.17 (2.33)	5.78 (1.73)	1.87 (1.62)	*p*<0.001 1, 2>3
Self-organization	18.24 (2.76)	8.90 (3.29)	6.00 (3.43)	*p*<0.001 1>2, 3; 2>3
Affect dysregulation	5.39 (1.76)	3.22 (1.99)	1.60 (1.49)	*p*<0.001 1>2, 3; 2>3
Negative self-concept	6.45 (1.46)	2.21 (1.64)	2.03 (1.86)	*p*<0.001 1>2, 3
Interpersonal problems	6.40 (1.52)	3.47 (1.78)	2.37 (1.64)	*p*<0.001 1>2, 3; 2>3
Functional impairment	2.75 (0.49)	2.35 (0.42)	2.15 (0.36)	*p*<0.001 1>2, 3; 2>3

### Type of trauma as predictor of class status

Logistic regression analyses were performed to determine whether the type of trauma was predictive of diagnostic status. Participant identification of childhood abuse as the worst trauma was a significant predictor of complex PTSD as compared to PTSD (χ^2^(1)=5.23, *p*=0.022) and marginally predictive of complex PTSD as compared to any other class (χ^2^(1)=3.69, *p*<0.055). The odds ratio in the former analysis (OR= 2.11, 95% CI: 1.11, 3.99) indicates that individuals who reported childhood abuse as their worst trauma were nearly twice as likely to have complex PTSD as compared to PTSD. Conversely, participant identification of 9/11 as their worst trauma was a significant predictor of PTSD when compared to complex PTSD (χ^2^(1)=13.56, *p*<0.001) as well as when compared to any other class (χ^2^(1)=15.38, *p*<0.001). The odds ratio in the former analysis (OR= 4.05, 95% CI: 1.92, 8.52) indicates that individuals who reported 9/11 as their worst trauma were four times as likely to have PTSD as compared to complex PTSD (See Table 6).

**Table 6:** Trauma history as predictor of class

Predictor	Class comparisons	Beta (SE)	Odds ratio (95% CI)	*p*-Value
Childhood abuse as worst trauma	Complex PTSD vs. PTSD	0.37 (0.16)	2.11 (1.11, 3.99)	0.022
Childhood abuse as worst trauma	Complex PTSD vs. all others^a^	0.25 (0.13)	1.67 (0.99, 2.80)	0.055
9/11 as worst trauma	PTSD vs. complex PTSD	0.70 (0.19)	4.05 (1.92, 8.52)	< 0.001
9/11 as worst trauma	PTSD vs. all others^b^	0.59 (0.15)	3.27 (1.81, 5.90)	< 0.001

a All others=both of the alternative classes (PTSD and low symptoms)

b all others=both of the alternative classes (complex PTSD and low symptoms).

### Predictors of functional impairment

A hierarchical linear regression analysis was performed to determine the relative contribution of all four factors of complex PTSD to functional impairment. After controlling for age and gender, PTSD symptoms were predictive of functional impairment (*T*(278)=7.34, *p*<0.001) and the model as a whole was significant (*F*(3, 278)=20.48, *p*<0.001) with a total of 18.1% variance accounted for. The addition of the three factors unique to complex PTSD (i.e., affect dysregulation, negative-self concept, interpersonal problems) contributed an additional 21.2% of the explained variance and improved the model with a total 39.3% of the variance explained (*F*(6, 275)=29.66, *p*<0.001). Parameter estimates for both models are summarized in Table 7.

**Table 7:** Hierarchical linear regressions predicting functional impairment by four factors of complex PTSD

Variable	Unstandardized beta	Standard error	Standardized beta
Model 1			
Intercept	2.08	0.14	–
Age	<0.01	<0.01	0.09
Gender	−0.18	0.09	−0.11*
PTSD	0.03	0.01	0.40**
Model 2			
Intercept	1.76	0.13	–
Age	<0.01	<0.01	0.10*
Gender	−0.12	0.08	−0.07
PTSD	0.01	0.01	0.15*
Affect dysregulation	0.04	0.01	0.18**
Negative self-concept	0.04	0.01	0.23**
Interpersonal problems	0.05	0.01	0.23**

*Notes*: *n*=282 for both models due to missing data; females are the reference group in the “Gender” variable. Therefore, a significant negative value for this beta coefficient indicates that males have greater functional impairment

* *p*<0.05

** *p*<0.01.

### Exploratory analyses testing the model with individuals with BPD

**Fig. 3:**
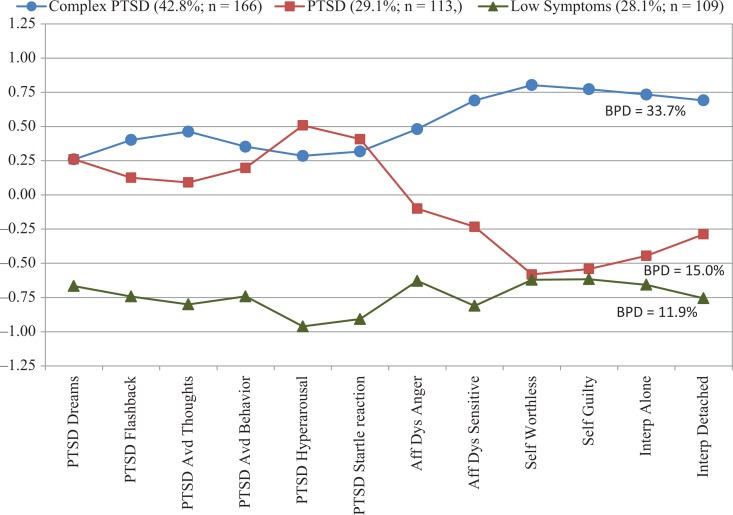
Mean standardized values of complex PTSD items with individuals with borderline personality disorder.

An LPA was repeated which included the individuals identified with BPD. The results of this analysis were nearly identical to the original LPA. The three-class model provided the best fit for the data and was selected over the two-class model as the BIC value was lower. The mean standardized values of the items for the complex PTSD, PTSD, and low symptom classes are shown in Fig. 3. The mean probability of class membership in the three-class model was acceptable: 0.96 for the complex PTSD class, 0.86 for the PTSD class, and 0.95 for the low symptom class, which implies acceptable discrimination among the classes. An acceptable entropy value probability of 0.83 lends support to this result by suggesting adequate latent class separation. Overall, 42.8% (*n*=166) of participants were classified into complex PTSD class, 29.1% (*n*=113) into the PTSD class, and 28.1% (*n*=109) into the low symptom class.

The proportion of individuals with BPD within each class was: 33.7% (*n*=56) of the complex PTSD class, 15.0% (*n*=17) of the PTSD class, and 11.9% (*n*=13) of the low symptom class. The sociodemographics, frequencies for types of trauma history and symptom severity characteristics of each of the classes resulting from two LPA analyses did not differ. ANOVAs were conducted to assess differences in symptom severity across the three classes. Significance values and the pattern of results of the pair-wise comparison tests were identical to those observed in the first LPA. With the exception that avoidance symptoms were significantly higher in the complex PTSD class when compared to the PTSD class, while in the original LPA this was not a significant difference. Still, the two classes did not differ on PTSD total symptom severity.

### Discussion

The study assessed the ICD-11 proposal to organize traumatic stress diagnoses into two distinct disorders, PTSD and complex PTSD, by evaluating the validity of the diagnoses using LPA. The LPA identified that subgroups of treatment-seeking individuals were empirically distinghuishable based on different patterns of symptom endorsement. In a treatment-seeking sample of trauma-exposed adults, a three-class model was identified: a class high in PTSD symptoms, as well as in affective, negative self-concept, and interpersonal problems (complex PTSD), a class high in PTSD but low in the other symptoms (PTSD) and a class that was low in all symptoms (low symptom). Childhood abuse was predictive of complex PTSD compared to PTSD and conversely 9/11 exposure was predictive of PTSD compared to complex PTSD. Finally, the complex PTSD class experienced greater functional impairment than the PTSD class.

The analyses assessing the relationship of trauma history to class support the use of trauma history as an identified risk factor and may guide the clinician in making a differential diagnosis between complex PTSD and PTSD but the data suggest, consistent with Courtois (2004), that history is not determinative of diagnosis. Notably, a small group of participants who identified 9/11 as their worst trauma (20%) fell into the complex PTSD class and 23% of those who identified childhood abuse as their worst trauma fell into the PTSD group, suggesting the probabilistic rather than determinative nature of history as a guide to diagnosis. These data are consistent with and support the ICD proposal that the differential diagnosis of PTSD and complex PTSD are symptom-based rather than history-based disorders. Finally, a substantial proportion of individuals in the low symptom class had experienced 9/11 exposure (indeed, in numbers similar to those in the PTSD group) or childhood abuse (again in numbers not dramatically less than those in the complex PTSD class). These individuals appear relatively healthy, suggesting that they represent a “resilient” subgroup about whom further analysis regarding personal and environmental characteristics (e.g., sprituality, social support) might lead to some insight about resilience.

In summary, the different symptom profiles that describe PTSD and complex PTSD are associated with different subgroups of individuals, different levels of impairment, and different risk factors (trauma history). These data provide evidence supporting the ICD-11 proposal for two distinct disorders, a classification organization that will facilitate clinician identification of the symptom profiles. This approach contrasts with that of the DSM-5 proposal for PTSD which has expanded the diagnosis to include symptoms related to affect dysregulation and negative self-concept (e.g., see Criteria D and E and the specifier or subtype for dissociation). The formulation of a multi-cluster, multi-symptom disorder diagnosis with specifiers/subtype is inconsistent with the notion of clinical utility, particularly on a global level. International surveys have indicated that mental health providers prefer diagnoses to have a limited number of symptoms and tend to disregard subtype/specifier information (Reed, Correia, Esparza, Saxena, & Maj, 2011). The proposal to have PTSD and complex PTSD side-by-side as sibling disorders is responsive to clinician preferences and consistent with the overall ICD-11 classification plan for mental disorders to be presented in a “flatter” horizontal structure rather than vertical.

The organization of trauma-related problems into two disorders, PTSD and complex PTSD may have more clinical utility than the DSM-5 proposal of PTSD in several ways. This categorization scheme may be superior in regards to implementation characteristics. Implementation characteristics include factors such as ease of recall and use, goodness of fit (accuracy of description for any one patient) and time required to use the diagnosis. In addition the characteristics which distinguish the two ICD-11 disorders have substantial clinical relevance. Differences in risk factors (trauma history) provide an easy “rule of thumb” to help guide diagnosis and differences in the level of impairment have implications for clinical management. The distinction between PTSD and complex PTSD may help organize clinical services in an effective and efficient way, particularly with regard to the selection of interventions and the duration of treatment.

There is substantial empirical literature suggesting that PTSD can be resolved in short-term (9–12 weeks) trauma-focused interventions (see Foa, Keane, Friedman, & Cohen, 2008). A longer course of treatment might be necessary for the effective treatment of complex PTSD, where treatment would include resolving greater numbers and types of problems and addressing more severe functional impairment. There are several therapies which have been developed and tested for the complex PTSD symptoms defined above or variations thereof and would include interventions directly attending to affect dysregulation difficulties, relational and social difficulties, and directly or indirectly engaging in exercises to support the reorganization of a more positive and compassionate self-concept (see Cloitre et al., 2012). The relative benefits of shorter versus longer and multi-targeted therapies for both PTSD and complex PTSD remain to be determined.

Some concern has been expressed about the overlap of symptoms that occurs between the complex PTSD and BPD diagnoses (e.g., Resick et al., 2012). From a clinical utility perspective, the disorders are quite distinct. Complex PTSD focuses on the effects of trauma, has PTSD symptoms as a core element of the disorder, and is associated with a treatment plan that includes the relatively rapid treatment of PTSD symptoms through trauma-focused interventions. The most salient and clinically relevant features of BPD are high risk of suicide, suicide attempts and self-injurious behavior and the diagnosis and its effective treatment has been organized around these issues (Linehan, 1993). In addition, the nature of self-concept and interpersonal difficulites in BPD emphasize problems with a lack of a stable self-concept and fears of abandonment. In contrast, complex PTSD is defined by the presence of a stable negative self-concept and avoidance of relationships. These differences have significant implications for treatment. BPD is likely to require a longer course of treatment and particular attention to the task of termination as the therapy draws to a close. Nevertheless, one important test of the discriminability of the two disorders is whether, in fact, the symptoms of the disorders describe different and distinct classes of individuals. LPA similar to those used in this study can be applied to determine whether the complex PTSD and BPD symptom profiles describe different classes of patients but this requires a different and larger data set than the one used in this study. Such an investigation is ongoing.

The analyses conducted in this study focused on evaluating the integrity and coherence of the complex PTSD diagnosis. The confirmatory factor analysis proposing a four-factor complex PTSD diagnosis fits the data well and supported the coherence of the construct in the absence of BPD. The LPA analyses were conducted both without and with individuals with BPD and the class groupings did not change nor did the symptom profiles, suggesting the stability of both PTSD and complex PTSD with and without comorbid BPD (see Figs. 2 and 3). Finally, BPD was found to co-occur among all three classes but the proportion varied within each class, suggesting its independence as a disorder. In this sample, BPD occurred in the absence of both PTSD and complex PTSD (i.e., in the low symptom group) as well as co-occurred, at different rates, with the other two disorders. The co-occurrence of complex PTSD and BPD is no more or less acceptable than other comorbidities observed in psychiatry, and more generally in medicine, where comorbid disorders can share overlapping symptoms (e.g., high blood pressure in obesity and artherosclerosis). The clinical utility of having two identified disorders with some shared symptoms may be determined by the presence of recognizable differences in their core features, differences in the prognosis, and in the treatment plan.

In summary, this study provides preliminary evidence of the validity of the ICD-11 proposal for the distinct, but related diagnoses of PTSD and complex PTSD. However, there are several limitations that should be kept in mind. First, the study is preliminary in that it uses archival data from which to construct the diagnosis of PTSD and complex PTSD. The current analyses do not include items focused on dissociation and difficulty in experiencing positive affect, both of which were not available in this database. The exemplar symptoms of the core features may change, pending repeated tests of the constucts. The study was completed in a clinic that specialized in interpersonal violence and the sample was predominately female. Replication of the LPA results in different settings, with reference to different stressors, and with different populations around the world is necessary. The development and testing of self-report and clinical interview measures of ICD-11 PTSD and omplex PTSD are important next steps. In addition, studies comparing the differences between complex PTSD and BPD are important.

Finally, it should be noted that careful thought has been given and empirical evidence has been provided regarding a variety of definitions and classification options for PTSD, PTSD with a dissociative subtype, complex PTSD, and dissociative disorders (see, e.g., Lanius, Brand, Vermetten, Frewen, & Spiegel, 2012; Sar, 2011) and that no single option is the inevitable “right answer.” Continued study and conversation about these different diagnostic approaches is important and will require the identification of priorities regarding the purpose of disease classification including clinical utility, scientific advance, and resource allocation.

## Conclusion

LPA identified the presence of three different classes of trauma-exposed individuals that support the proposed ICD-11 distinction between PTSD and complex PTSD. This classification approach to traumatic stress disorders has the benefit of conforming to the ICD-11 classification goals of clinical utility by virtue of its relative simplicity in the classification structure, clear differences in conceptual organization, and limited set of symptom features. In addition, noted differences in risk factors and level of impairment may contribute, respectively, to ease of diagnosis and treatment management decisions, both important characteristics of clinical utility.

## Disclaimer

M. Cloitre, C. R. Brewin, R. A. Bryant, and A. Maercker are members of the WHO of the Working Group on the Classification of Stress-Related Disorders. However, the views expressed reflect the opinions of the authors and not necessarily the Working Group and the content of this article does not represent WHO policy.
